# Serological and molecular prevalence of canine vector-borne diseases (CVBDs) in Korea

**DOI:** 10.1186/s13071-017-2076-x

**Published:** 2017-03-16

**Authors:** Guk-Hyun Suh, Kyu-Sung Ahn, Jong-Ho Ahn, Ha-Jung Kim, Christian Leutenegger, SungShik Shin

**Affiliations:** 1 Department of Internal Medicine, College of Veterinary Medicine, Chonnam National University, Gwangju, South Korea; 2 Department of Parasitology, Animal Medical Institute, College of Veterinary Medicine, Chonnam National University, 77 Yongbong-ro, Gwangju, 61186 South Korea; 3 IDEXX Laboratories, Inc., Westbrook, ME 04092 USA

**Keywords:** Canine vector-borne diseases (CVBD), Korea, Real-time PCR, Haemotropic mycoplasma, Serology, Prevalence

## Abstract

**Background:**

Previous surveys in dogs from Korea indicated that dogs are exposed to a variety of vector- borne pathogens, but perception for a nation-wide canine vector-borne disease (CVBD) occurrence has been missing. We report here results of both serological and molecular prevalence studies for major CVBDs of dogs from all over the South Korean Peninsula except for Jeju Island.

**Results:**

Serological survey of 532 outdoor dogs revealed the highest prevalence for *Dirofilaria immitis* (25.2%), followed by *Anaplasma phagocytophilum* (15.6%), *Ehrlichia canis* (4.7%) whereas *Borrelia burgdorferi* showed the lowest prevalence (1.1%). The number of serologically positive dogs for any of the four pathogens was 216 (40.6%). Concurrent real-time PCR assay of 440 dogs in the study indicated that DNA of “*Candidatus* M. haematoparvum”, *Mycoplasma haemocanis*, *Babesia gibsoni*, *A. phagocytophilum*, and *Hepatozoon canis* was identified in 190 (43.2%), 168 (38.2%), 23 (5.2%), 10 (2.3%) and 1 (0.2%) dogs, respectively. DNA of *Bartonella* spp., *Ehrlichia* spp., *Leishmania* spp., *Rickettsia* spp. and *Neorickettsia risticii* was not identified. Analysis of questionnaires collected from owners of 440 dogs showed that the number of dogs with heartworm preventive medication was 348 (79.1%) among which dogs still positive to *D. immitis* infection were 60 (17.2%), probably due to the mean months of heartworm preventive medication being only 6.5. The high prevalence rates of both “*Ca.* M. haematoparvum” and *Mycoplasma haemocanis* in dogs from Korea indicate that these organisms may be transmitted by vectors other than *Rhipicephalus sanguineus* because this tick species has rarely been found in Korea. This is the first nationwide survey for canine haemotropic mycoplasma infections in Korea.

**Conclusions:**

This study showed that the risk of exposure to major vector-borne diseases in dogs is quite high throughout all areas of South Korean Peninsula. Since achieving full elimination of many pathogens causing CVBDs from infected animals is often impossible even when they are clinically cured, dogs once exposed to CVBDs can remain as lifetime reservoirs of disease for both other animals and humans in the close vicinity, and should therefore be treated with preventative medications to minimise the risk of pathogen transmission by the competent vectors.

## Background

Previously, we reported results of a serological survey for *Dirofilaria immitis*, *Anaplasma phagocytophilum*, *Ehrlichia canis* and *Borrelia burgdorferi* infections in rural hunting and urban shelter dogs mainly from south-western regions of the Republic of Korea [1] in which the highest prevalence observed was for *D. immitis* (22.3%), followed by *A. phagocytophilum* (18.8%), *E. canis* (6.1%) and the lowest prevalence was for *B. burgdorferi* (2.2%) among 229 hunting dogs. In contrast, stray dogs found within the city limits of Gwangju showed seropositivity only to *D. immitis* (14.6%) and none of the 692 dogs responded positive for *A. phagocytophilum*, *E. canis* or *B. burgdorferi* antibodies.

Although our previous survey in dogs showed that they are exposed to multiple vector-borne pathogens, perception for a nation-wide canine vector-borne disease (CVBD) occurrence has been missing. Haemotropic mycoplasmas, for instance, are a group of bacteria that parasitize the surface of erythrocytes in a wide range of mammal species including dogs which are mainly infected with *Mycoplasma haemocanis* and “*Candidatus* Mycoplasma haematoparvum”. Infections are usually chronic and subclinical in immunocompetent dogs but may lead to clinical signs related to haemolytic anaemia following splenectomy, immunosuppression or concurrent infections [2]. Although the natural mode of transmission of feline and canine haemoplasmas has not been definitely elucidated, blood transfusions and blood-sucking arthropods such as ticks have been implicated to be involved in the transmission of haemoplasmas in dogs and cats [3–5]. Since dogs in Korea are expected to be frequently exposed to arthropod infestation during their outdoor activities, they are also vulnerable to the infection with haemotropic mycoplasmas.

This article reports results of a serological survey on selected vector-borne diseases in dogs from five major provinces of the South Korean Peninsula. Also, a molecular survey on arthropod-borne pathogens such as “*Ca.* M. haematoparvum”, *M. haemocanis*, *Babesia* spp., *Hepatozoon* spp., *Bartonella* spp., *Leishmania* spp., *Neorickettsia* spp. and *Rickettsia* spp. were included in the survey.

## Methods

**Fig. 1:**
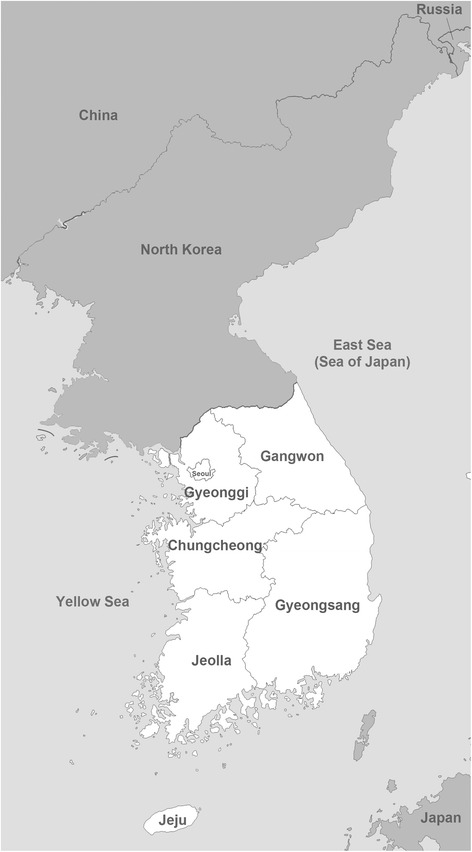
Distribution map showing the location of the 5 major provincial divisions in the Republic of Korea. Jeju Island was not included in this study

From January of 2012 to December of 2016, blood samples were collected from 532 dogs from all over the South Korean Peninsula which included 100 dogs from Chungcheong, 35 dogs from Gangwon, 88 dogs from Gyeonggi, 123 dogs from Gyeongsang and 186 dogs from Jeolla provincial areas (Fig. 1). All dogs included in this study were strictly outdoor dogs at an average of 3.2 years of age with slightly higher number of male dogs (300/532, 56.4%). The majority of dogs were of mixed-bred (48.1%) followed by Korean native Jindo (17.7%), laikas (14.3%), hounds (7.0%), pointers (4.9%) and terriers (2.4%).

Blood samples were collected from the cephalic or lateral saphenous veins of dogs and were stored in EDTA tubes. Blood samples were tested within 30 min after collection using a commercial ELISA assay kit (SNAP® 4Dx®; IDEXX Laboratories, Inc., Westbrook, ME, USA) which detected *D. immitis* antigen, and antibodies specific to *Anaplasma* spp. [*A. phagocytophilum* and *A. platys*, synthetic peptide from the major surface protein (p44/MSP2)], *Ehrlichia* spp. (*E. canis* and *E. ewingii*, P30 and P30-1 outer membrane proteins), and *B. burgdorferi* (C6 peptide).

Of 532 dogs in the survey, blood samples from 440 dogs were also tested by real-time PCR assays which included a canine haemotropic Mycoplasma test (RealPCR test code #2632, IDEXX Laboratories, Inc., West Sacramento, CA, USA) and a Tick/Vector canine comprehensive panel with Lab 4Dx (test code #2871, IDEXX Laboratories, Inc.). Hydrolysis probe based real-time PCR assays were designed and validated according to published protocols [6]. Pathogens of interest included in the study were *A. phagocytophilum* (msp2 [p44], GenBank accession number of target gene: DQ519570), *A. platys* (groEL, heat-shock protein, AY848753), *Babesia* spp. (ssrRNA, AF271082), *Bartonella* spp. (citrate synthase gene, AJ439406), *Mycoplasma haemocanis* (ribosomal RNA ssrRNA, AF197337), “*Ca.* Mycoplasma haematoparvum” (ribosomal RNA ssrRNA, AY383241), *Ehrlichia canis* (disulfide oxidoreductase, dsb, gene, AF403710), *E. ewingii* (dsb gene, AY428950), *E. chaffeensis* (dsb gene, AF403711), *Hepatozoon canis* (ssrRNA, AF176835), *H. americanum* (ssrRNA, AF176836), *Leishmania* spp. (glycoprotein gp63, YO8156), *Neorickettsia risticii* (ribosomal RNA 16S RNA, AF184082), and *Rickettsia rickettsii* (GroEL heat-shock protein, AJ293326). Samples positive by PCR for *Babesia* spp. were submitted for species-specific real-time PCR testing, including *B. canis* (heat-shock protein, hsp 70, AB248735), *B. canis vogeli* (hsp 70, EF527401), *B. canis rossi* (hsp 70, AB248738), *B. felis* (ITS-2, AY965742), *B. gibsoni* (hsp 70, AB248731), and *B. conradae* (ITS-2, AY965742 [7]. In addition, for confirmatory sequencing purposes, two outside primers beyond the synthetic positive control were designed, normally leading to a 300 to 500 bp long PCR product. These re-sequencing outside primers were used to confirm the analytical specificity of the real-time PCR tests and to resolve discrepant results.

Real-time PCR was performed as single plex reactions with six quality controls, including quantitative PCR-positive controls (synthetic DNA, Integrated DNA Technologies IDT, Coralville, IA, USA), PCR-negative controls (RNase-free PCR-grade water, Fisher Scientific, Waltham, MA, USA), negative extraction controls (lysis solution only), quantitative DNA internal sample quality control targeting the host 18S rRNA gene complex, an internal positive control spiked into the lysis solution, and an environmental contamination monitoring control (swab-based laboratory monitoring). Real-time PCR tests were validated analytically and clinically according to industry standard protocols (Applied Biosystems, User Bulletin #3 [8]), in order to obtain fully standardised real-time PCR tests. Following the industry standard allowed for the design and validation of standardized real-time PCR tests which could be carried out in parallel as a panel. For the analytical validation, each assay was evaluated for six validation criteria including amplification efficiency, linearity, reproducibility intra-run, reproducibility inter-run, 2 square value, and signal-to-noise ratio of the fluorescent signal.

Briefly, 90 μl of blood was lysed in a guanidinium thiocyanate-based lysis solution, incubated for 10 min and extracted using Whatman filters on a Corbett X-Tractor platform (Qiagen, Valencia, CA, USA). Nucleic acids were eluted into 150 μl of PCR-grade nuclease-free water (Fisher Scientific, Fremont, CA, USA) and 5 μl amplified in subsequent single-plex real-time PCR reactions. Analysis was performed on a Roche LightCycler 480 (Roche Applied Science, Indianapolis, USA) and raw data analysed using the 2nd derivative maximum method to generate crossing points (CP).

A test of independence for significance of the relationship between categorical variables (gender, age, and geographic regions) for both serological and molecular studies was made via Pearson’s Chi-square test and Fisher’s exact test for expected counts under five using SPSS 17.0 (SPSS Inc., Chicago, IL, USA).

Questionnaires were delivered to owners of the 440 dogs for which both 4Dx and RealPCR assays were tested. The questionnaire was designed in order to obtain information as to whether their dogs were periodically medicated with heartworm preventive compounds and parasiticides.

## Results

The serological prevalence of *D. immitis*, *Anaplasma* spp., *Ehrlichia* spp*.* and *B. burgdorferi* in 532 outdoor dogs from Korea is shown in Table 1. The number of dogs serologically positive to any of the four pathogens surveyed in this study was 216 (40.6%). The number of dogs with single, dual, triple or quadruple seropositivity was 188 (35.3%), 26 (4.9%), 0 (0.0%) and 2 (0.4%), respectively. The highest prevalence was observed for *D. immitis* (134, 25.2%), followed by *Anaplasma* spp. (83, 15.6%), *Ehrlichia* spp. (25, 4.7%); the lowest prevalence was recorded for *B. burgdorferi* (6, 1.1%).

**Table 1:** Seroprevalence of selected arthropod-borne pathogens in outdoor dogs from five major provincial areas of South Korea

Category		Dogs examined (%)	Number(%) of positive dogs by SNAP 4Dx test					
			Di Ag	Ap Ab	Ec Ab	Bb Ab	Cumulative total	Total^a^
Gender	Female	300 (56.4)	64 (12.0)	31 (5.8)	7 (1.3)	2 (0.4)	104 (19.5)	95 (17.9)
	Male	232 (43.6)	70 (13.2)	52 (9.8)	18 (3.4)	4 (0.8)	144 (27.1)	121 (22.7)
Age (yrs)	< 2	115 (21.6)	23 (4.3)	15 (2.8)	2 (0.4)	0 (0.0)	40 (7.5)	36 (6.8)
	≥ 2	417 (78.4)	111 (20.9)	68 (12.8)	23 (4.3)	6 (1.1)	208 (39.1)	180 (33.8)
Geographical origin (provincial areas)	Chungcheong	100 (18.8)	26 (4.9)	18 (3.4)	4 (0.8)	1 (0.2)	49 (9.2)	45 (8.5)
	Gangwon	35 (6.6)	1 (0.2)	3 (0.6)	1 (0.2)	0 (0.0)	5 (0.9)	5 (0.9)
	Gyeonggi	88 (16.5)	20 (3.8)	21 (3.9)	7 (1.3)	1 (0.2)	49 (9.2)	39 (7.3)
	Gyeongsang	123 (23.1)	14 (2.6)	27 (5.1)	7 (1.3)	4 (0.8)	52 (9.8)	41 (7.7)
	Jeolla	186 (35.0)	73 (13.7)	14 (2.6)	6 (1.1)	0 (0.0)	93 (17.5)	86 (16.2)
Total		532 (100)	134 (25.2)	83 (15.6)	25 (4.7)	6 (1.1)	248 (46.6)	216 (40.6)

*Abbreviations*: *Di Ag* *Dirofilaria immitis* antigen, *Ap Ab* *Anaplasma phagocytophilum* antibody, *Ec Ab* *Ehrlichia canis* antibody, *Bb Ab* *Borrelia burgdorferi* antibody

^a^Number of positive dogs by any of the four test results by SNAP 4Dx test

Seropositive dogs to any of the four pathogens was higher in male dogs (22.7%) than in female dogs (17.9%; *χ* ^2^ = 22.7720, *P <* 0.001), and dogs of above 2 years were significantly more exposed to these pathogens than younger dogs (34, 6%; *χ* ^2^ = 6.3525, *P* < 0.012). Geographically, dogs from Gangwon and Jeolla provincial areas were significantly more exposed to any of the four selected vector-borne pathogens than the rest of the regions of South Korea (*χ* ^2^ = 10.7582 and 3.7655, respectively; *P* = 0.001 and 0.052, respectively). Mixed breed dogs had lower prevalence for *D. immitis* infection than other breeds (15.2 *vs* 34.4%; *χ* ^2^ = 25.9441, *P* < 0.001) whereas Jindo dogs had higher prevalence for *D. immitis* than other breeds (57.4 *vs* 18.3%; *χ* ^2^ = 63.0665, *P* < 0.001). Similarly, mixed breed dogs had lower in prevalence for any four of the pathogens detected by SNAP 4Dx test (32.4 *vs* 48.2%; *χ* ^2^ = 13.6896, *P* < 0.001) whereas Jindo dogs had higher prevalence than other breeds (58.5 *vs* 36.8%; *χ* ^2^ = 15.1844, *P* < 0.001).

Results of the molecular prevalence study of 440 dogs for 10 selected vector-borne pathogens by real-time PCR are shown in Table 2. Although most dogs (62.3%) were positive to at least one of the 10 pathogens, the most commonly identified were “*Ca.* M. haematoparvum” (43.2%) and *Mycoplasma haemocanis* (38.2%), the prevalence of which were significantly higher among male dogs (*χ* ^2^ = 20.2113 and 11.2841, respectively, both *P* < 0.001). The number of dogs positive to both “*Ca.* M. haematoparvum” and *M. haemocanis* was 95 (21.6%), while the number of dogs positive to any of the two mycoplasmas was 263 (59.8%). Compared to other breeds, higher prevalence rates were observed in mixed-breed dogs for both “*Ca.* M. haematoparvum” (54.7 *vs* 27.2%) and *Mycoplasma haemocanis* (41.4 *vs* 33.7%) infections, but significant difference was only observed for “*Ca.* M. haematoparvum” (*χ* ^2^ = 33.0302, *P* < 0.001 *vs χ* ^2^ = 2.6965, *P* < 0.1006).

**Table 2:** Molecular prevalence of selected arthropod-borne pathogens by real-time PCR in outdoor dogs from five major provincial areas of South Korea

Category		Dogs examined (%)	Number(%) of positive dogs by real-time PCR											
			CMh	Mh	Bg	Ap	Hc	Br	Eh	Lsh	Nr	Ri	Total	Any PCR Total^a^
Gender	Female	183 (41.6)	56 (12.7)	53 (12.0)	9 (2.0)	5 (1.1)	1 (0.2)	0 (0.0)	0 (0.0)	0 (0.0)	0 (0.0)	0 (0.0)	57 (13.0)	89 (20.2)
	Male	257 (58.4	134 (30.5)	115 (26.1)	14 (3.2)	5 (1.1)	0 (0.0)	0 (0.0)	0 (0.1)	0 (0.2)	0 (0.3)	0 (0.4)	106 (24.1)	185 (42.0)
Age (yrs)	< 2	87 (19.8)	9 (2.0)	28 (6.4)	0 (0.0)	3 (0.7)	1 (0.2)	0 (0.0)	0 (0.1)	0 (0.2)	0 (0.3)	0 (0.4)	41 (9.3)	36 (8.2)
	≥ 2	353 (80.2)	181 (41.1)	140 (31.8)	23 (5.2)	7 (1.6)	0 (0.0)	0 (0.1)	0 (0.2)	0 (0.3)	0 (0.4)	0 (0.5)	351 (79.8)	238 (54.1)
Geographical origin (provincial areas)	Chungcheong	100 (22.7)	22 (5.0)	21 (4.8)	1 (0.2)	2 (0.5)	0 (0.0)	0 (0.1)	0 (0.2)	0 (0.3)	0 (0.4)	0 (0.5)	46 (10.5)	38 (8.6)
	Gangwon	35 (8.0)	21 (4.8)	13 (3.0)	2 (0.5)	0 (0.0)	0 (0.0)	0 (0.0)	0 (0.0)	0 (0.0)	0 (0.0)	0 (0.0)	36 (8.2)	28 (6.4)
	Gyeonggi	88 (20.0)	33 (7.5)	29 (6.6)	10 (2.3)	1 (0.2)	1 (0.2)	0 (0.0)	0 (0.0)	0 (0.0)	0 (0.0)	0 (0.0)	74 (16.8)	46 (10.5)
	Gyeongsang	123 (28.0)	65 (14.8)	65 (14.8)	1 (0.2)	4 (0.9)	0 (0.0)	0 (0.0)	0 (0.0)	0 (0.0)	0 (0.0)	0 (0.0)	135 (30.7)	92 (20.9)
	Jeolla	94 (21.4)	49 (11.1)	40 (9.1)	9 (2.0)	3 (0.7)	0 (0.0)	0 (0.0)	0 (0.0)	0 (0.0)	0 (0.0)	0 (0.0)	101 (23.0)	70 (15.9)
Total		440 (100.0)	190 (43.2)	168 (38.2)	23 (5.2)	10 (2.3)	1 (0.2)	0 (0.0)	0 (0.0)	0 (0.0)	0 (0.0)	0 (0.0)	392 (89.1)	274 (62.3)

*Abbreviations*: *CMh* “*Candidatus* M. haematoparvum”, *Mh* *Mycoplasma haemocanis*, *Bg* *Babesia gibsoni*, *Ap* *Anaplasma phagocytophilum*, *Hc* *Heptatozoon canis*, *Br* *Bartonella* spp., *Eh* *Ehrlichia* spp., *Lsh* *Leishmania* spp., *Nr* *Neorickettsia* spp., *Ri* *Rickettsia* spp

^a^Number of positive dogs by any of the ten test results by real-time PCR test

Of those 440 dogs for which both real-time PCR and SNAP 4Dx tests were performed, 83 dogs were serologically positive to *Anaplasma* spp. while only 10 dogs (2.3%) were real-time PCR-positive; the species was identified as *A. phagocytophilum*. The number of dogs positive by both serological and real-time PCR to *Anaplasma* spp. was 6 (1.4%) while the number of dogs positive to *Anaplasma* spp. by either serological or real-time PCR methods was 87 (19.8%). None of the 25 dogs serologically positive to *Ehrlichia* spp. were positive by real-time PCR method. Dogs positive to *Babesia gibsoni* (5.2%), *A. phagocytophilum* (2.3%) and *Hepatozoon canis* (0.2%) were few, and no dogs were positive to *Bartonella* spp., *Ehrlichia* spp., *Leishmania* spp., *Neorickettsia* spp. or *Rickettsia* spp.

Of those 440 dogs for which both real-time PCR and SNAP 4Dx tests were performed, the number of dogs on heartworm preventive medication, as responded by their owners, was 348 (79.1%). The number of dogs with heartworm medication but still positive to *D. immitis* infection was 60 (13.6%). However, the mean number of months that these dogs were on heartworm medication was only 6.5 months. Owners of 292 dogs (66.4%) responded that they medicated their dogs with both heartworm preventives and yearly parasiticides. Owners of 332 dogs (75.5%) responded that they visit veterinary clinics at an average of 2.02 times per year.

## Discussion

Previously, we did a similar serological survey from December of 2007 to August of 2009 for *D. immitis*, *A. phagocytophilum*, *E. canis* and *B. burgdorferi* infections in rural hunting and urban shelter dogs from southwestern regions of South Korea using IDEXX SNAP 4Dx kit [1]. Considering that the 532 dogs in this study were all outdoor dogs and from all over South Korea, the results showed CVBD patterns similar to those in hunting dogs from the south-western regions of South Korea.

It was surprising that 60 out of 440 dogs (13.6%) were on heartworm preventive medication but still seropositive to *D. immitis*. As 348 dog owners responded that their dogs were on heartworm preventive medication, 17.2% of these dogs were not protected by the preventive medication. Although the Korean Peninsula has a temperate climate with four distinct seasons (hot and humid summer; cold and dry winter; and mild and pleasant spring and fall), adults of some species of mosquitoes are active throughout the year including freezing winter months [9, 10]. Therefore, our result clearly show that all year round heartworm preventive medication is required for a successful prevention of dogs from heartworm disease in Korea. Considering that the mean number of months these dogs were on heartworm preventive medication was only 6.5 while 75.5% of dog owners visited their veterinary clinics at an average of 2.02 times per year, it appears that education for both veterinary practitioners and dog owners for a strict compliance to year round heartworm preventive medication is critical for a successful prevention of heartworm disease in Korea.

Compared to other vector-borne pathogens surveyed in this study, dogs were highly exposed to the two major haemotropic mycoplasmas of dogs, “*Ca.* M. haematoparvum” (43.2%) and *M. haemocanis* (38.2%). Since all dogs investigated in this study were clinically healthy, the two haemotropic mycoplasmas affecting dogs from Korea may cause chronic or asymptomatic infections, as is the case in other countries unless dogs are either splenectomised or immune-compromised [11–13]. Since previous reports indicate that canine haemoplasma infection has been associated with immune-mediated haemolytic anaemia (IMHA) [14, 15], the potential clinical significance of this finding needs to be further investigated in the future in relation to the causation of IMHA by haemoplasmas in dogs in South Korea. Significantly higher infection rates of the two mycoplasmas among male dogs in this study coincide with previous reports on the prevalence of canine hemotropic mycoplasma infections in Mediterranean countries [16]. This is the first nation-wide survey for canine haemotropic mycoplasma infections in Korea

Although the natural mode of transmission of feline and canine haemoplasmas has not been definitely elucidated, blood transfusion, blood-sucking arthropods and more recently mange mites may be involved [11]. Among tick species, *Rhipicephalus sanguineus* and *Ixodes* spp. have been indicated as the major vectors for canine haemotropic mycoplasmas, but different transmission patterns may be present in Korea because very low population of *Rhipicephalus* and *Ixodes* spp. infests dogs in Korea [17]. Instead, majority of ticks found in dogs from Korea is either *Haemaphysalis longicornis* or *H. flava* which indicates that the principle arthropod vectors transmitting canine haematrophic mycoplasmas in Korea may be different from those in other countries. Since the risk of exposure to canine haemoplasmosis among outdoor dogs from Korea have been shown to be high, both pet owners and veterinary practitioners should be alerted in Korea.

Our study indicates that considerably high number of dogs were also exposed to *B. gibsoni* infection (5.2%). Unlike other countries where dogs are exposed to more than one species of *Babesia*, *B. gibsoni* is the only known causative agent for canine babesiosis in Korea [18]. As in other countries, high infection rates among pit bull terriers were also reported in Korea [19].

Of those 440 dogs for which both real-time PCR and SNAP 4Dx tests were performed, discrepancy was observed between the exposure rates of *Anaplasma* spp. by serological assay (18.9%) and by real-time PCR (2.3%). A previous report from Germany indicates that no significant differences were observed concerning the rates of seropositivity or PCR-positivity in dogs with and without clinical signs of canine granulocytic anaplasmosis [20]. However, if a survey is performed in an area endemic for anaplasmosis seropositivity rates outnumber PCR-positivity. For example, in a study of 731 dogs from Minnesota, USA, the serological survey using the IDEXX SNAP 4Dx test as in our study among both healthy and clinically anaplasmosis-suspected dogs showed high seropositive response to *A. phagocytophilum* (67.4 and 53.7% respectively, 55.4% combined), while only clinically anaplasmosis-suspected dogs were highly positive by the PCR assay (37.3%). Among the healthy group of dogs, however, only 7 of 222 dogs (3.2%) were PCR-positive to *A. phaocytophilum* [21].

One dog from Gyeonggi Province showed positive to *Hepatozoon canis* by real-time PCR. Although four free-ranging Korean leopard cats (*Prionailurus bengalensis*) have been previously reported to contain schizonts of *H. felis* in the heart muscle, there has been no report of *H. canis* infection among dogs from Korea. The dog in our study was an 18 month-old female mixed-breed dog lactating a litter of four puppies. Since this was the first indication of *H. canis* infection in dogs in Korea, attempts were made to trace back the dog for a morphological identification of the organism, but in vain because the dog was killed by a car accident before the revisit.

## Conclusions

This study clearly shows that outdoor dogs from all over the South Korea are frequently exposed to pathogens causing major CVBDs. We also report for the first time that haemotropic mycoplasma infections with *M. haemocanis* and “*Ca.* M. haematoparvum” are especially high among these dogs. Since achieving full elimination of the pathogens from the animal is often impossible even when achieving a ‘clinical cure’, treated dogs can remain as reservoirs of disease for dogs or other animals and humans in the close vicinity, and should therefore be treated with preventative compounds to minimise the risk of pathogen transmission by the competent vectors.
